# Effect of Repeated Consumption of Partially Hydrolyzed Guar Gum on Fecal Characteristics and Gut Microbiota: A Randomized, Double-Blind, Placebo-Controlled, and Parallel-Group Clinical Trial

**DOI:** 10.3390/nu11092170

**Published:** 2019-09-10

**Authors:** Zenta Yasukawa, Ryo Inoue, Makoto Ozeki, Tsutomu Okubo, Tomohisa Takagi, Akira Honda, Yuji Naito

**Affiliations:** 1 Nutrition Division, Taiyo Kagaku Co., Ltd., Yokkaichi, Mie 510-0844, Japan; 2 Academic-Industrial Graduate School, Mie University, Tsu, Mie 514-8507, Japan; 3 Laboratory of Animal Science, Department of Agricultural and Life Sciences, Kyoto Prefectural University, Kyoto 606-8522, Japan; 4 Molecular Gastroenterology and Hepatology, Graduate School of Medical Science, Kyoto Prefectural University of Medicine, Kyoto 602-8566, Japan; 5 Gastroenterology, Tokyo Medical University Ibaraki Medical Center, Inashiki, Ibaraki 300-0395, Japan

**Keywords:** partially hydrolyzed guar gum, Bristol stool scale, diarrhea, gut microbiota, 16S rRNA

## Abstract

Partially hydrolyzed guar gum (PHGG) is a water-soluble dietary fiber and is used in solid and liquid food to regulate gut function. The aim of this study was to investigate effects of PHGG on bowel movements (stool form and frequency), plasma bile acids, quality of life, and gut microbiota of healthy volunteers with a tendency toward diarrhea, i.e., irritable bowel syndrome diarrhea (IBS-D)-like symptoms. A randomized, double-blind, placebo-controlled, and parallel trial was performed on 44 healthy volunteers (22 males, 22 females, 41.9 ± 6.3 years old (average ± SD)) with minimum 7 bowel movements every week, wherein above 50% of their stool was between the Bristol stool scale (BSS) value of 5 and 6. Intake of the PHGG for 3 months significantly improved stool form, evaluated using BSS, and had no effects on stool frequency. BSS was significantly normalized in the group consuming the PHGG compared with the placebo. Comprehensive fecal microbiome analysis by the 16S rRNA-sequence method detected significant changes in the ratio of some bacteria, such as an increase of *Bifidobacterium* (*p* < 0.05) in the PHGG group. Our results suggest that intake of PHGG improves human stool form via regulating intestinal microbiota.

## 1. Introduction

Increasing evidence demonstrates the bidirectional interactions within the gut-brain axis [1]. Alterations in these interactions have not only been implicated in functional gastrointestinal disorders, such as irritable bowel syndrome (IBS) [2,3], but also in psychiatric and neurologic pathologies including affective disorders [4,5], autism spectrum disorders (ASD) [4,6], Parkinson’s disease [7], multiple sclerosis [8], and chronic pain [9].

Gastrointestinal symptom, including diarrhea/soft stool distress is associated with worse mental and physical health-related quality of life [10]. Diarrhea could be problematic not only in case of diseases and treatments, but also in non-disease status [11,12,13,14,15,16].

Recently, we showed the profiles of stool consistency using the Bristol stool scale (BSS) along with a fecal microbiome analysis for healthy Japanese adults and the ratio of subjects with BSS type 5 or type 6 were relatively high (31.1%) in all male subjects [17].

Dietary fiber’s action on intestinal environment has attracted considerable attention [18] and water-soluble dietary fiber, such as PHGG, inulin, and indigestible dextrin, action on bowel movement has been reported [19,20,21]. However, few studies have examined the effects of dietary fibers on loose stool tendency (IBS-Diarrhea-like symptoms).

PHGG is a water-soluble dietary fiber derived from the endosperm of *Cyamopsis tetragonolobus* L. seeds, mainly composed of galactose and mannose with approximately a 1:2 ratio. PHGG has been in the market as a dietary fiber for nearly three decades under the trade name Sunfiber^®^.

PHGG has multiple benefits, for example prebiotic effects [22,23] with the production of high amounts of short chain fatty acids [23,24,25]. PHGG is also found to be effective in lowering hyperglycemia [26,27] and hyperlipidemia [28,29] and it helps to maintain satiety [30].

As for bowel habits, PHGG consumption led to a favorable impact on constipation prevention as revealed by systematic review along with the application of meta-analysis [19]. In addition, PHGG could reduce or treat diarrhea in several patient studies [31,32,33,34]. However, as far as we know, PHGG’s effect on healthy subject diarrhea was tested only in one study, in which hyperosmotic transitory diarrhea was induced by ingesting non-digestible sugar alcohols [35].

In the present study, we evaluated and compared the effect of PHGG on diarrhea symptoms to that of a placebo using a randomized double-blind design. In addition, as secondary outcomes, microbiota were assessed.

## 2. Materials and Methods

### 2.1. Study Design

This was a randomized, double-blind, placebo-controlled, and parallel-group study carried out in a single clinical center (Medical Corporation K. Medical Office TOC Building Clinic, Tokyo) in Japan. Kyoto Prefectural University of Medicine and Taiyo Kagaku Co., Ltd. (TKC, Mie, Japan) prepared the study protocol together and TKC provided the study products. All study procedures were undertaken by a clinical research organization (imeQ Inc. (IMI) Tokyo, Japan) on consignment from TKC.

### 2.2. Ethical Approval of the Study Protocol

This study was conducted according to the guidelines laid down in the Declaration of Helsinki and all procedures involving human subjects were approved by SUDA Clinic Institutional Review Board (Tokyo, Japan). Written informed consent was obtained from all subjects. The study is registered on the University hospital Medical Information Network Clinical Trials Registry (UMINCTR: http://www.umin.ac.jp/ctr/index.htm) as number UMIN000030184 (the direct link to registered study information; https://upload.umin.ac.jp/cgi-open-bin/ctr_e/ctr_view.cgi?recptno¼R000034461).

### 2.3. Study Products

The commercial PHGG (Partially hydrolyzed guar gum, guar fiber, and galactomannan fiber) preparation (Sunfiber^®^) used in this study was provided by TKC. The PHGG is manufactured by treatment of guar gum with a *β*-endogalactomannase from a strain of *Aspergillus niger*.

PHGG relieves IBS symptoms in patients at 5–11 g/day [36]. The PHGG dose was determined to be 5 g/day (as 4 g total dietary fiber by enzymatic-gravimetric-liquid chromatographic method; 3.5 g total dietary fiber by enzymatic-gravimetric method). The placebo was maltodextrin (Dextrose equivalence 10.0–12.0).

### 2.4. Subjects

Eighty-eight healthy Japanese male and female volunteers (20–49 years) were selected from volunteers registered in IMI. Then, they gave written informed consent, detailed their medical history, and underwent tests (physiological, biochemical, hematological). Each of these physiological, biochemical, and hematological tests were performed at the Showa Medical Science Cor. (Tokyo, Japan). Volunteers who did not meet the exclusion criteria and also met inclusion criteria were enrolled in 1-week screening. Selected volunteers recorded their fecal frequency in a diary and the BSS score (described in the Data collection section below). Then, volunteers who had a lower stool consistency score were excluded. Finally, 44 volunteers were selected as study subjects. The inclusion criterion was subjects who were healthy with a loose stool (minimum 7 bowel movements every week, wherein above 50% of their stool was between the BSS values of 5 and 6). There were 15 exclusion criteria. The first exclusion criterion was patients likely to have inflammatory bowel disease, gastrointestinal ulcers, pancreatitis, coeliac disease, lactose intolerance, protozoan infection, parasitic infection, other organic gastrointestinal disease or pregnant women. The second criterion was subjects using commercially available drugs, “quasi-drugs”, or dietary supplements for the prevention and treatment of diarrhea with abdominal pain and abdominal discomfort on a daily basis. The third criterion was patients currently attending hospital for diarrhea treatment who had pain and discomfort in the abdomen. The fourth criterion was patients judged by the principal investigator (P. I.) to require medical treatment because of severe diarrhea with pain and discomfort in the abdomen. The fifth criterion was patients diagnosed with IBS. The sixth criterion was patients who had undergone gastrointestinal surgery (excluding appendectomy). The seventh criterion was women who were breastfeeding or pregnant, or who were planning to become pregnant during the study period. The eighth criterion was patients treated for a systemic disease. The ninth criterion was patients using non-steroidal anti-inflammatory drugs, corticosteroids, or antibiotics on a daily basis. The tenth criterion was subjects who were current smokers (except former smokers who had stopped smoking for at least a 1 year prior). The 11th criterion was subjects with an abnormal blood test (excluding those in which the range of physiological variation was acceptable according to the P. I.). The twelfth criterion was subjects who had participated in a clinical trial 60 d before the screening test. The thirteenth criterion was patients suffering from a mental illness. The penultimate exclusion criterion was subjects who might suffer an allergic reaction to the test food. The final exclusion criterion was subjects who were judged by the P. I. or by a collaborator to be unfit for this test.

### 2.5. Randomization

Subjects were randomly allocated into the PHGG group or the placebo group to balance for sex, age, fecal frequency, and BSS. Allocation was operated by a researcher of IMI who was not involved in taking measurements and analysis.

### 2.6. Blinding

TKC produced study products filled in aluminum sachets so that they were identical in appearance and flavor and provided them to IMI with a coded name. Correspondence of the coded name and the true name of the product was disclosed from TKC to IMI after completion of the data analyses.

### 2.7. Study Protocol

**Figure 1:**
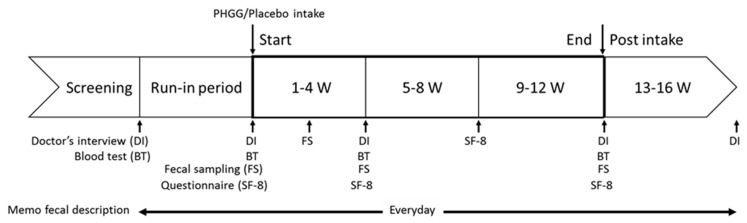
Test schedule.

After a 4-week run-in period, 22 participants were allocated to the PHGG group and the other 22 participants were allocated to the placebo group. Then, each participant ingested 1 sachet/d for 12 weeks (1–12 W). This schedule was followed by a 4-week washout period (13–16 W). After the end of the trial, participants who had experienced adverse events underwent follow-up examination until the disappearance of the events. Participants recorded their life survey and defecation questionnaire in a diary every day during the study period. At the end of run-in (0 W), after 4 weeks consumption (4 W), and after 12 weeks consumption (12 W) and washout periods (16 W), they visited the clinical center and detailed their symptoms. They provided blood and urine for biochemical/hematological tests and urinalyses at the end of run-in (0 W), after 4 weeks consumption (4 W), and after 12 weeks consumption (12 W). They also answered the Short Form-8 (SF-8) every month. The test schedules are summarized in Figure 1. Restrictions on participants during the study were avoidance of (i) an “irregular” lifestyle (lack of sleep, overeating, excessive drinking, poor diet); (ii) consumption of dietary supplements (except for the test sachets); and (iii) smoking. Additionally, participants were asked to maintain habitual diets and retain the quantity/quality of exercise and foods and the quantity of sleep throughout the study period.

### 2.8. Data Collection

The primary endpoints were defecation questionnaires (stool form and frequency). The secondary endpoints were gut microbiota, serum bile acids, and SF-8.

Fecal frequency was assessed by recording defecation times each day. Fecal characteristics were assessed at each defecation using the Bristol Stool Scale, as follows: Value of 1 (separate hard lumps, like nuts), 2 (“sausage-shaped” but lumpy), 3 (like a sausage but with cracks on its surface), 4 (like a sausage or snake, smooth and soft), 5 (soft blobs with clear-cut edges), 6 (fluffy pieces with rugged edges, a mushy stool), and 7 (watery, no solid pieces). Serum bile acids were analyzed by the liquid chromatography-tandem mass spectrometry (LC-MS/MS) method, as described previously [37]. General QoL (quality of life) was assessed using the SF-8. At the end of each period, participants were asked eight questions about their health status in the past month. Then, a mental-component summary score and a physical component summary score were calculated as described previously [38].

### 2.9. Fecal Sample Collection and DNA Extraction

Fecal samples were collected and gut bacterial composition analysis was performed. Fecal samples, the size of a grain of rice, were collected using a guanidine thiocyanate solution (Feces Collection kit; Techno Suruga Lab, Shizuoka, Japan). After vigorous mixing, the samples were stored at a temperature not higher than room temperature for a maximum of 7 days until DNA extraction. Genomic DNA was isolated using the NucleoSpin microbial DNA kit (MACHEREY–NAGEL, Düren, Germany). Approximately 500 μL of the stored fecal sample was placed in a microcentrifuge tube containing 100 μL of elution buffer (BE). The mixture was then placed into a NucleoSpin bead tube with proteinase K and subjected to beating with mechanical beads for 12 min at 30 Hz in the TissueLyser (TL). The subsequent extraction procedure was performed per the manufacturer’s instructions. Extracted DNA samples were purified using the Agencourt AMPure XP (Beckman Coulter, Brea, CA, USA).

### 2.10. Sequencing of 16S rRNA Gene

Two-step polymerase chain reactions (PCRs) were performed for the purified DNA samples to obtain sequence libraries. The first PCR was performed to amplify and used a 16S (V3–V4) metagenomic library construction kit for NGS (Takara Bio Inc, Kusatsu, Japan) with primer pairs 341F (50-TCGTCGGCAGCGTCAGATGTGTATAAGA GACAGCCTACGGGNGGCWGCAG-30) and 806R (50-GT CTCGTGGGCTCGGAGATGTGTATAAGAGACAGGGA CTACHVGGGTWTCTAAT-30) corresponding to the V3–V4 region of the 16S rRNA gene. The second PCR was performed to add the index sequences for the Illumina sequencer with a barcode sequence using the Nextera XT index kit (Illumina, San Diego, CA, USA). The prepared libraries were subjected to sequencing of 250 paired-end bases using the MiSeq Reagent v3 kit and the MiSeq (Illumina) at the Biomedical Center at Takara Bio.

### 2.11. Microbiome Analysis

The processing of sequence data, including chimera check, operational taxonomic unit (OTU) definition, and taxonomy assignment, was performed using QIIME version 1.9, USEARCH version 8.0, and UCHIME version 4.2.40, according to the work of Inoue et al. [39]. Singletons were removed in this study. Taxonomy assignment of the resulting OTU was completed using RDP classifier version 2.10.2 and the Greengenes database (published May 2013). Statistical differences (*p* < 0.05) in the relative abundance of bacterial genera (>1.0%) between groups were evaluated using two-way ANOVA.

### 2.12. Safety Assessment

The P. I. assessed the safety of study products based on the results of participant communication, tests (physiological, biochemical, hematological), and urinalyses. The content of the daily diary was also referred to for safety assessment.

### 2.13. Sample Size

Although PHGG was tested on IBS patients, there is no testing on these healthy subjects, so we were unable to estimate the minimum number of subjects. Then, we set the minimum number of subjects to be 20 for statistical analysis. We also recruited 2 extra participants for each treatment in case of dropouts. Overall, 44 subjects (22 in each group) were enrolled in this study.

### 2.14. Statistical Analysis

Statistical analyses were done as per the protocol set using R version 3.1.1 with *p* < 0.05 (two tailed). Intra-group comparison was done with the paired Dunnett at each week for all outcomes. Inter-group comparisons of treatment were made using Welch’s *t* test. The subjects with BSS scores 4 were compared by Chi-square test between groups. A two-way repeated-measures ANOVA, with PHGG or placebo, and a time interaction factor was examined to evaluate the interaction effects on the outcome measures. Both ANOVA and power analyses were conducted using JMP software version 11.2.0 (SAS institute).

Defecation questionnaires (fecal frequency and characteristics) were recorded daily. Then, fecal frequency at each week and mean stool characteristics (BSS 1–7) at each week were compared between groups. Intra-group comparison with the baseline at each week was also undertaken.

Stool volume, abdominal pain, urgency, abdominal discomfort, distension, incomplete evacuation, straining, passage of gas, and borborygmi were calculated as the mean value for 1 week. Then, the values were compared between groups. Intra-group comparison with the baseline at each week was also carried out. An intergroup comparison of the raw scores of the SF-8 was also done at the end of each period.

## 3. Results

### 3.1. Subjects and Compliance

**Figure 2:**
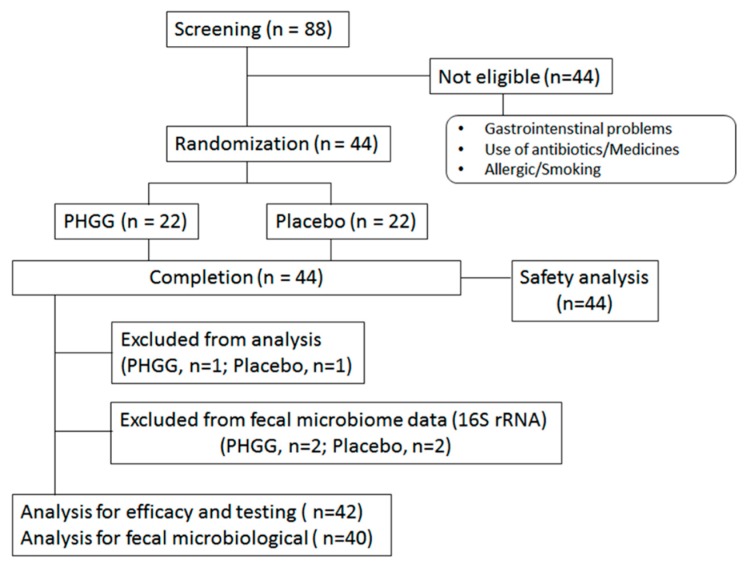
Flow chart of study participants.

This study was performed from January–June 2018. Eighty-eight volunteers were initially screened (Figure 2). According to the described inclusion/exclusion criterion, 44 were excluded from the study due to lower stool consistency scores and non-consistent parameters. The finally selected 44 volunteers were randomly allocated into the groups to receive PHGG or the placebo.

During the study period, no subjects dropped out from the study. As a result, 22 volunteers in the PHGG group and 22 volunteers in the placebo group completed the study. The prevalence of entries in the daily diary and sachet consumption in participants who completed the study was 100% and 99.6%, respectively. Therefore, all 44 subjects met the inclusion criteria for statistical analyses. Two participants used antibiotics during chronic study period and were excluded from loose stool efficacy analysis (PHGG group, n = 1; Placebo group, n = 1). Two additional participants used antibiotics during the stool sampling period, so these plus the previous two subjects were excluded from the fecal microbiome analysis (PHGG group, n = 2; Placebo group, n = 2). As a result, 44 subjects were evaluated for safety analysis, 42 subjects were evaluated for loose stool efficacy analysis, and 40 subjects were evaluated for fecal microbiome analysis.

The baseline characteristics of the participants are shown in Table 1. There were no significant differences between the PHGG and the placebo group for any baseline characteristic (*p* ≥ 0.05).

### 3.2. Gastrointestinal-Related Function

**Figure 3:**
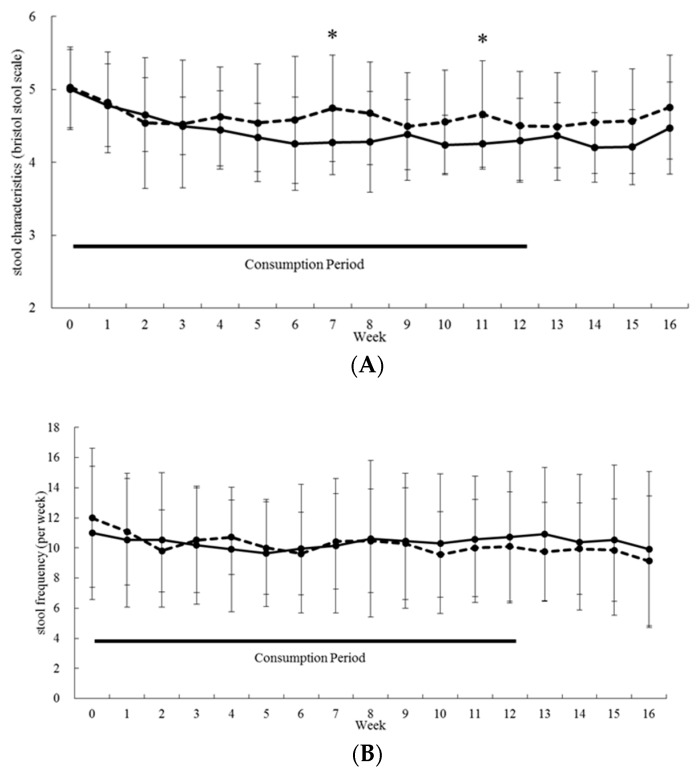
Effect of PHGG on primary endpoints. Stool form (**A**) and frequency (**B**) are shown. Straight lines and dotted lines denote mean values for PHGG and placebo groups, respectively. Bars show SD. PHGG group: n = 21, placebo group: n = 21. * *p* < 0.05, Welch’s *t*-test.

We set stool form and frequency as primary endpoints in this study. As shown in Figure 3A, stool form was significantly lower from 3 W to 16 W compared with baseline in the PHGG group (*p* < 0.01). The PHGG group also showed a significantly (*p* < 0.05) lower BSS compared with the placebo group at 7 W and 11 W (power analysis values estimated was 0.70 and 0.61, respectively). A two-way repeated measures ANOVA revealed a significant interaction between time and PHGG treatment (*p* < 0.05).

As PHGG significantly normalized BSS (close to score 4), we further analyzed this effect by calculating percentage of subjects with improved fecal characteristics data at or above 50% of the BSS at level 4. PHGG consumption at 5, 7, and 11 weeks significantly increased the percentage (Figure S1). There were no significant inter-group differences at any time point concerning stool frequency (Figure 3B).

There were no significant inter-group or intragroup differences on stool volume, abdominal pain, urgency to defecate, abdominal discomfort, distension/bloating, incomplete evacuation, straining, passage of gas, and borborygmi throughout consumption and washout periods (data not shown).

### 3.3. Quality of Life

In QoL evaluation using SF-8, there were no significant inter-group differences in the physical-component summary (Table 2) or physical related subgroup (physical function, role physical, bodily pain, and general health) (Table S1).

As for mental-related items, social functioning at 8 W and 12 W had a significant difference and the emotional role at 4 W and mental health at 4 W tended to be higher in the PHGG group (Table S1). As a result, mental-component summaries at 4 W tended to be higher in the PHGG group (Table 2).

### 3.4. Fecal Microbiological and Serum Bile Acid Analysis

**Figure 4:**
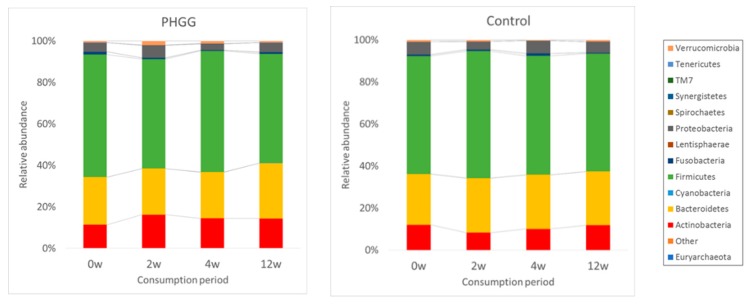
Comparative analyses of the taxonomic composition of the microbial community at the phylum level for each group at each consumption period. Each component of the cumulative bar chart indicates a phylum.

As gut microbiota plays an important role on gastrointestinal function, we conducted fecal microbiome analysis by the 16S rRNA-sequence method. Across the 12 weeks of PHGG consumption, phylum Actinobacteria, accounting for more than 10% of total bacteria, increased significantly (*p* < 0.05) and differed significantly compared to the placebo (Figure 4). In the genus level, the abundance of *Bifidobacterium* belonging to phylum Actinobacteria was significantly increased in the PHGG group (*p* < 0.05) (Table 3) and differed significantly compared to the placebo during the consumption period. PHGG also increased the abundance of *Ruminococcus* and *Megasphaera*, while it reduced *Bacteroides*, the unclassified genus belonging to family Lachnospiraceae, and *Phascolarctobacterium* significantly between the groups (Table 3).

We conducted correlation analysis of these microbiota with fecal characteristics. *Bifidobacterium* abundance at 12 W was negatively correlated with fecal characteristics at 12 W (ρ = −0.31, *p* < 0.05). *Ruminococcus* abundance at 2 W and 4 W was negatively correlated with fecal characteristics at 16 W (ρ = −0.36, *p* < 0.05 and ρ = −0.35, *p* < 0.05, respectively). *Bacteroides* abundance at 2 W was positively correlated with fecal characteristics at 11 W and 12 W (ρ = 0.32, *p* < 0.05 and ρ = 0.35, *p* < 0.05, respectively). Unclassified genus belonging to family Lachnospiraceae abundance at 2 W was positively correlated with fecal characteristics from 3 W and 15 W (ρ > 0.32, *p* < 0.05 except for fecal characteristics from 3 W).

Bile acids (BAs) are transformed by gut microbiota and may affect gastrointestinal function, so we measured serum BA levels in series. Although, we did not detect any inter-group difference for all BAs measured, the conversion of primary BAs into secondary BAs calculated by deoxycholic acid/(cholic acid + deoxycholic acid) in serum decreased significantly in the PHGG group after 12 weeks (*p* < 0.05), but not in the placebo group (Table 4). We could not connect gastrointestinal function with serum BA levels from our results.

### 3.5. Adverse Events

Unusual events described by participants in the daily diary were constipation, diarrhea, cold, influenza type B, food poisoning, sprain, wound, bruise, abdominal pain, abdominal distension, abdominal discomfort, nasal inflammation, coughing, malaise, sore throat, lumbago, headache, stomach ache, nausea, neuralgia and sinusitis. These events were transient and incidental and were confirmed by the P. I. not to be correlated with consumption of test food.

## 4. Discussion

We investigated the effect of PHGG on diarrhea symptoms in healthy volunteers who had a trend of diarrhea. We demonstrated that PHGG improved fecal characteristics after continuous intake. Although the significance could be observed at certain timepoints (W7 and W11) compared to the placebo, the PHGG intake is considered clinically significant because BSS score could approach nearly 4.0 during 3–16 W compared to the baseline. In addition, the number of subjects above 50% of BSS at level 4 was higher in the PHGG group (Figure S1).

Many probiotics are available for food or medicine [40], but to our knowledge, no effective probiotics exist for IBS-D. Additionally, antimotility agents exist but the effects are limited [41]. Therefore, PHGG’s effect on IBS-D like symptoms could be considered important.

PHGG’s effect on diarrhea has been reported in several reports so far, mainly toward patients. PHGG’s effect on healthy subject diarrhea was tested on hyperosmotic transitory diarrhea induced by ingesting non-digestible sugar alcohols [35]. In this study, we firstly demonstrated PHGG’s effect on healthy volunteer’s diarrhea symptoms (not induced diarrhea) in a randomized double-blinded placebo controlled clinical trial. We previously showed the proportion of the Japanese population with a BSS of 5–6 accounted for about one fourth to two thirds [17], so PHGG is thought to be useful for populations who tend to have diarrhea.

We compared fecal microbiota composition between the PHGG and the placebo group after chronic intake and found PHGG increased the abundance of *Bifidobacterium*, *Ruminococcus*, and *Megasphaera*, while *Bacteroides*, an unclassified genus belonging to family Lachnospiraceae, and *Phascolarctobacterium* were significantly reduced in the PHGG group. Correlation analysis showed that stool consistency was negatively correlated with *Bifidobacterium* and *Ruminococcus* abundance and positively correlated with *Bacteroides* and the unclassified genus belonging to family Lachnospiraceae abundance. As stool consistency is reported to be associated with gut microbiota [42], these bacteria may be involved in stool normalization.

*Bifidobacterium* has been classically used for treating pediatric diarrhea [43] and has a suggested efficacy for prevention and treatment of pediatric antibiotic-associated diarrhea by a meta-analysis [44]. One strain of *Bacteroides*, *Bacteroides fragilis*, is known to be associated with diarrheal disease [45].

Qualitative changes in gut microbiota were also suggested to be associated with IBS. In IBS patients, lower amounts of *Bifidobacterium* and higher amounts of *Bacteroides* than healthy controls were reported [46,47]. In this study, we found an increased abundance of *Bifidobacterium* and a decreased abundance of *Bacteroides*. This result suggested that PHGG might have normalized gut microbiota to healthier compositions.

The administration of PHGG in IBS patients has been reported by several reports. Giaccari et al. [48] administered a balanced diet supplemented by PHGG (5 g/day) to 134 IBS patients and found a positive effect of PHGG on various IBS symptoms. Parisi et al. [49] investigated PHGG (5 g/day) in 188 IBS patients and compared it to a 12-week wheat bran diet (30 g/day of wheat bran). Improvements in core IBS symptoms were observed with both the PHGG and bran, but the former was better tolerated and preferred by patients. Parisi et al. [50] also published an open trial study in which they compared the effects of PHGG administered at two dosages (5 and 10 g/day) for 3 months in patients with IBS, followed by three months of follow-up. In these studies, microbiota change was not measured, but the last paper demonstrated improvement of IBS symptoms and QoL (SF-36) for the patients in both dosage groups. In our study, mental-related items, especially social functioning, were significantly improved by PHGG intake. As far as we know, the current study showed QoL improvement by PHGG intake in a controlled study for the first time.

So far, PHGG’s study toward fecal microbiota is limited. PHGG (6 g/day) was reported to increase the fecal *Bifidobacterium* and the butyrate producing bacteria after 2 weeks of intake in female university students using the real-time PCR method [51]. In another study, PHGG (21 g/day) was reported to increase the fecal *Bifidobacterium* spp. after 2 weeks of intake in healthy male volunteers by the culture method [22]. In our recent report, PHGG was administered to children with autism spectrum disorder and fecal microbiota change was monitored before and after PHGG administration by 16S rRNA metagenomics [52]. This study adds PHGG’s effect on microbiota and demonstrated a prebiotic effect toward healthy volunteers who tend to have diarrhea using 16S rRNA metagenomics.

We observed an increase in *Bifidobacterium*, *Ruminococcus*, and *Megasphaera* abundance with PHGG intake. In an earlier study, *Bifidobacterium* could not ferment PHGG in vitro [18], suggesting that stimulation of *Bifidobacterium* by PHGG would be due to the degraded products derived during PHGG degradation by other bacteria of the large intestine. It is still not clear that what kind of bacteria ferment PHGG, however considering that *Streptococcus* has an increased similarly with *Bifidobacterium*, it may degrade PHGG and supply degradation products for Bifidogenic activity.

In addition, *Ruminococcus* and *Megasphaera* increase with PHGG intake reveals that prebiotic PHGG may result in stimulating these bacteria. The reason for this is not yet clear, however it is hypothesized to be involved in butyrate production. Butyrate is considered to be an important stimulator for gut, which is involved in water absorption, restoring mucosal damage and atrophy, and increasing water absorption from the epithelium [53,54].

Diarrhea is defined as loose, mushy, or watery stools or a stool frequency of more than three bowel movements per day [55]. Therefore, accelerating water absorption in gut epithelia and suppressing bowel movement is very important. In this study, fecal characteristics were normalized by PHGG, but not affected the fecal frequency. Our results indicate that PHGG may help accelerate water absorption. Since *Ruminococcus* and *Megasphaera* are involved in butyrate production and butyrate accelerates water absorption, PHGG may act via butylate. PHGG metabolites, short chain fatty acids, and secondary bile acids are known to affect bowel movement and their involvement should be further investigated.

One limitation of the current study is a relatively small sample size and a single PHGG dose. A larger sample size and multiple doses should be tested in the future to further add to the literature.

## 5. Conclusions

In conclusion, this study demonstrated that PHGG normalized stool consistency, helping volunteers with loose stools get closer to a BSS score of 4. This effect may be modulated through gut microbiota changes, which may also improve mental QoL.

## Figures and Tables

**Table 1:** Background of the subjects at baseline.

	Total	PHGG	Placebo	*p* Value
Number of subjects (male; female)	44 (22; 22)	22 (11; 11)	22 (11; 11)	
Age (years)	41.9 ± 6.3	41.8 ± 7.0	42.1 ± 5.6	0.906
Height (cm)	166.7 ± 8.4	167.0 ± 6.2	166.3 ± 10.3	0.773
Weight (kg)	62.7 ± 10.9	63.4 ± 8.2	62.0 ± 13.2	0.685
BMI (kg m^−2^)	22.4 ± 2.5	22.7 ± 2.5	22.2 ± 2.6	0.511
Systolic blood pressure (mmHg)	109.5 ± 11.3	110.3 ± 11.2	108.6 ± 11.6	0.636
Diastolic blood pressure (mmHg)	64.3 ± 9.5	64.1 ± 9.1	64.6 ± 10.1	0.876
Pulse (per minute)	69.7 ± 9.1	69.8 ± 8.5	69.7 ± 10.0	0.974
Waist circumference (cm)	80.6 ± 8.1	81.6 ± 6.5	79.7 ± 9.5	0.446

Each value represents the mean ± S.D. Welch’s *t*-test (between groups).

**Table 2:** Physical and mental component summaries of quality of life, elucidated using the SF-8 questionnaire.

	Physical Component Summary		Mental Component Summary	
	PHGG	Placebo	PHGG	Placebo
Baseline (0 W)	50.65 ± 4.82	51.77 ± 4.56	50.87 ± 4.94	48.61 ± 7.52
	*p* = 0.443		*p* = 0.257	
Intake period 1 (4 W)	52.41 ± 4.34	52.79 ± 4.44	50.89 ± 4.65	47.15 ± 7.96
	*p* = 0.782		*p* = 0.072	
Intake period 2 (8 W)	51.13 ± 4.29	51.27 ± 5.03	51.53 ± 4.38	48.19 ± 8.54
	*p* = 0.920		*p* = 0.121	
Intake period 3 (12 W)	51.59 ± 3.88	52.12 ± 4.75	50.89 ± 3.64	47.84 ± 9.46
	*p* = 0.692		*p* = 0.179	

Welch’s *t*-test (between groups).

**Table 3:** Relative abundance of bacterial genera in the fecal microbiota of each group at each consumption period.

Phylum	Class	Oder	Family	Genus	Group	Consumption Period (Week)			
						0	2	4	12
*Actinobacteria*	*Actinobacteria*	*Bifidobacteriales*	*Bifidobacteriaceae*	*Bifidobacterium*	Placebo	8.82% ± 7.94%	5.65% ± 5.17%	7.14% ± 6.42%	7.98% ± 7.27%
					PHGG	8.02% ± 6.35%	12.24% ± 8.54%	10.96% ± 8.15%	11.40% ± 10.45%
*Bacteroidetes*	*Bacteroidia*	*Bacteroidales*	*Bacteroidaceae*	*Bacteroides*	Placebo	16.56% ± 9.82%	16.23% ± 8.94%	18.99% ± 10.35%	16.96% ± 10.55%
					PHGG	13.21% ± 7.69%	12.93% ± 6.16%	13.12% ± 8.80%	14.85% ± 9.23%
*Firmicutes*	*Bacilli*	*Lactobacillales*	*Streptococcaceae*	*Streptococcus*	Placebo	0.46% ± 0.40%	0.47% ± 0.31%	0.82% ± 0.91%	0.54% ± 0.95%
					PHGG	0.61% ± 0.47%	0.92% ± 1.44%	1.17% ± 1.65%	1.60% ± 2.17%
*Firmicutes*	*Clostridia*	*Clostridiales*	*Clostridiaceae*	*Clostridium*	Placebo	0.74% ± 0.95%	0.58% ± 0.56%	1.24% ± 3.15%	0.58% ± 0.62%
					PHGG	0.38% ± 0.44%	0.27% ± 0.23%	0.24% ± 0.26%	0.22% ± 0.22%
*Firmicutes*	*Clostridia*	*Clostridiales*	*Lachnospiraceae*	*Unclassified*	Placebo	6.41% ± 2.65%	7.56% ± 3.87%	6.76% ± 4.02%	6.54% ± 2.22%
					PHGG	5.56% ± 2.87%	4.96% ± 2.55%	5.26%± 3.03%	4.91% ± 2.85%
*Firmicutes*	*Clostridia*	*Clostridiales*	*Lachnospiraceae*	*[Ruminococcus]*	Placebo	3.84% ± 3.58%	4.02% ± 3.22%	4.57% ± 5.72%	3.71% ± 2.76%
					PHGG	2.93% ± 2.29%	3.08% ± 3.49%	2.57% ± 1.72%	2.35% ± 1.57%
*Firmicutes*	*Clostridia*	*Clostridiales*	*Ruminococcaceae*	*Ruminococcus*	Placebo	2.45% ± 2.64%	2.39% ± 3.18%	1.97% ±3.02%	2.53% ± 2.98%
					PHGG	4.05% ± 4.06%	3.08% ± 4.40%	4.43% ± 4.94%	3.77% ± 4.86%
*Firmicutes*	*Clostridia*	*Clostridiales*	*Veillonellaceae*	*Megasphaera*	Placebo	0.74% ± 2.34%	0.54% ± 1.68%	0.89% ± 2.77%	0.90% ± 3.66%
					PHGG	2.52% ± 3.70%	2.97% ± 3.65%	3.41% ± 4.89%	3.75% ± 4.94%
*Firmicutes*	*Clostridia*	*Clostridiales*	*Veillonellaceae*	*Phascolarctobacterium*	Placebo	3.36% ± 2.96%	4.85% ± 4.38%	3.23% ± 2.77%	4.13% ± 4.18%
					PHGG	2.18% ± 2.29%	3.06% ± 3.22%	1.94% ± 2.05%	2.20% ± 2.63%

Data are expressed as the mean ± SD. Only the taxonomy of which the mean relative abundance (>1.0%) significantly differed between groups is listed. The relative abundance which showed a significant difference between groups at the baseline are excluded.

**Table 4:** Change in plasma bile acids after PHGG intake or placebo in healthy volunteers having a tendency toward diarrhea for 12 weeks.

	PHGG (n = 21)			Placebo (n = 21)			
Outcome	Initial	Final	Within Group *p* Value	Initial	Final	Within Group *p* Value	Between Groups *p* Value
Bile Acids Primary							
CA %	8.29 ± 4.96	8.63 ± 5.34	0.71	7.82 ± 5.30	8.79 ± 6.77	0.41	0.94
CDCA %	11.21 ± 10.71	13.11 ± 12.02	0.37	7.39 ± 6.03	10.92 ± 8.57	0.05	0.51
Bile Acids Secondary							
DCA %	16.79 ± 11.41	13.74 ± 12.38	0.23	14.20 ± 10.64	14.37 ± 11.03	0.92	0.87
LCA %	1.34 ± 0.91	1.25 ± 1.24	0.70	1.40 ± 1.17	1.19 ± 0.96	0.27	0.85
DCA/(CA+DCA)	0.6081 ± 0.2343	0.5108 ± 0.2685	0.03*	0.5798 ± 0.2370	0.5771 ± 0.2596	0.91	0.43
LCA/(CDCA+LCA)	0.2009 ± 0.1982	0.1840 ± 0.2354	0.71	0.2301 ± 0.2345	0.1762 ± 0.2072	0.29	0.91

CA, cholic acid; CDCA, chenodeoxycholic acid; DCA, deoxycholic acid; LCA, lithocholic acid. a Values are mean ± SD.

## Supplementary Materials

The following are available online at https://www.mdpi.com/2072-6643/11/9/2170/s1, Figure S1: Variation in percentage of subjects with improved fecal characteristics data minded at above 50% of Bristol Stool Score (BSS) at level 4., Table S1: Comprehensive statistics of the SF-8 scores of QoL questionnaire from respondents (n = 42).
