# Supplementary material for: Effect of Repeated Consumption of Partially Hydrolyzed Guar Gum on Fecal Characteristics and Gut Microbiota: A Randomized, Double-Blind, Placebo-Controlled, and Parallel-Group Clinical Trial
Source: Nutrients. 2019 Sep 10;11(9):2170. doi: 10.3390/nu11092170 (PMC6769658; doi:10.3390/nu11092170)
Supplement: Supplementary file 1 [file nutrients-11-02170-s001.pdf]

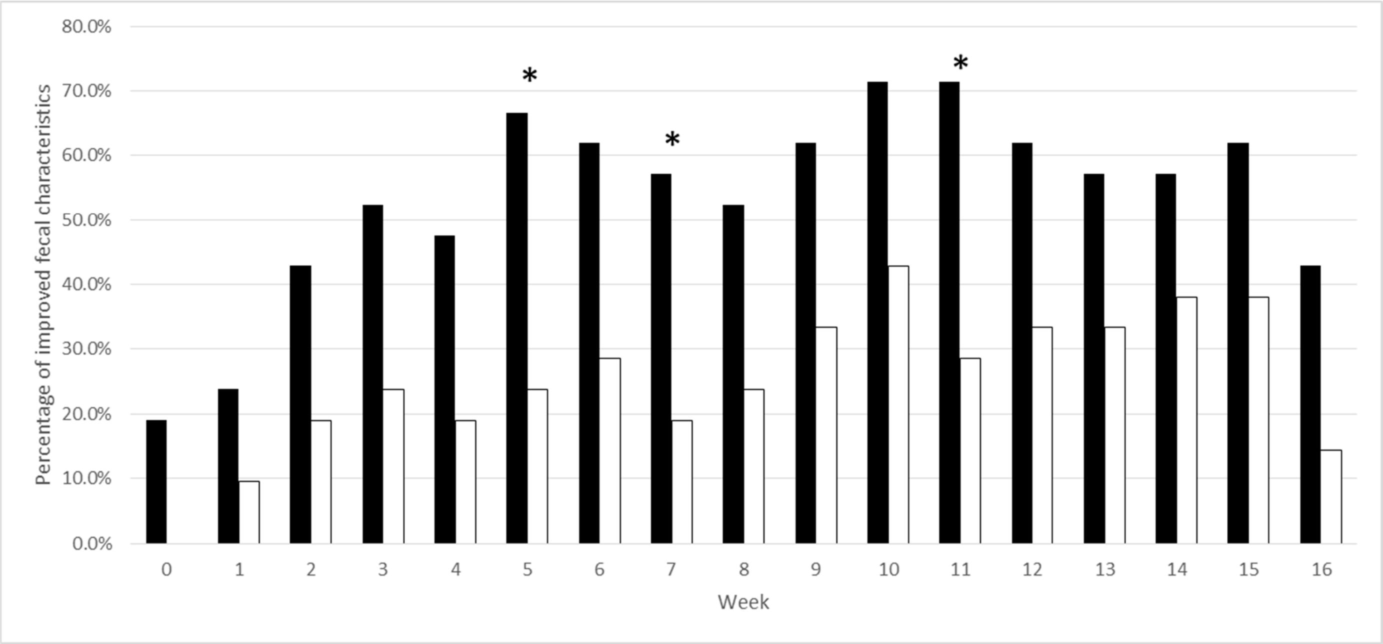

Figure S1. Variation in percentage of subjects with improved fecal characteristics data minded at above 50% of Bristol Stool Score (BSS) at level 4. Solid bars and open bars denote mean values for PHGG and placebo groups, respectively. PHGG group: n=21, placebo group: n=21. (*p<0.05, Chi-square test.).

Table S1. Comprehensive statistics of the SF-8 scores of QOL-questionnaire from respondents (n = 42).

	**Physical function**		**Role physical**		**Bodily pain**		**General health**		**Vitality**		**Social functioning**		**Role emotional**		**Mental health**	
	**PHGG**	**Placebo**	**PHGG**	**Placebo**	**PHGG**	**Placebo**	**PHGG**	**Placebo**	**PHGG**	**Placebo**	**PHGG**	**Placebo**	**PHGG**	**Placebo**	**PHGG**	**Placebo**
Baseline (0 W)	51.59±3.41	51.59±3.41	52.18±3.09	51.87±3.22	50.92±7.89	51.08±7.86	53.57±5.42	53.41±5.68	50.97±5.76	51.56±6.34	52.04±5.28	52.11±5.88	51.00±4.10	49.86±4.71	52.26±4.24	48.92±7.90
	p=1.000		p=0.746		p=0.949		p=0.924		p=0.751		p=0.965		p=0.409		p=0.098	
Intake period 1 (4 W)	52.41±3.05	52.41±3.05	51.86±3.87	51.86±3.87	56.51±5.87	54.17±7.54	53.74±5.14	51.92±5.71	51.25±5.24	50.82±6.21	52.44±5.11	49.99±7.18	52.17±3.96	49.35±6.00	52.87±4.80	49.08±7.71
	p=1.000		p=1.000		p=0.269		p=0.286		p=0.811		p=0.202		p=0.080		p=0.064	
Intake period 2 (8 W)	51.87±3.32	50.74±4.05	52.18±3.75	50.59±4.55	53.65±6.84	52.51±8.43	52.87±5.78	54.20±5.76	50.97±6.19	50.68±6.22	53.40±4.61	49.16±7.56	51.59±4.08	48.85±7.27	53.15±4.48	50.36±8.25
	p=0.331		p=0.224		p=0.633		p=0.461		p=0.883		p=0.035		p=0.142		p=0.183	
Intake period 3 (12 W)	52.44±2.32	51.29±3.99	51.22±4.53	51.54±4.49	53.13±5.87	52.13±6.73	54.53±5.19	54.59±5.76	50.81±5.40	51.28±6.79	53.78±3.42	49.97±7.68	51.29±4.10	49.11±6.86	51.96±4.11	49.37±8.65
	p=0.262		p=0.821		p=0.613		p=0.969		p=0.806		p=0.047		p=0.219		p=0.225	

Welch's t-test (between groups).
